# Musculoskeletal Ultrasonography Assessment of Functional Magnetic Stimulation on the Effect of Glenohumeral Subluxation in Acute Poststroke Hemiplegic Patients

**DOI:** 10.1155/2018/6085961

**Published:** 2018-07-03

**Authors:** Chengyuan Yang, Ping Chen, Wenjie Du, Qingmei Chen, Huilin Yang, Min Su

**Affiliations:** ^1^Department of Orthopedics, The First Affiliated Hospital of Soochow University, Suzhou, China; ^2^Department of Physical Medicine & Rehabilitation, The First Affiliated Hospital of Soochow University, Suzhou, China; ^3^Department of Physical Medicine & Rehabilitation, Wuxi Tongren International Rehabilitation Hospital, Wuxi, China

## Abstract

**Background:**

Glenohumeral subluxation (GHS) is common in patients with acute hemiplegia caused by stroke. GHS and upper limb function are closely related.

**Objective:**

Using musculoskeletal ultrasonography (MSUS) to objectively evaluate the efficacy of functional magnetic stimulation (FMS) in the treatment of GHS in acute hemiplegic patients after stroke.

**Methods:**

The study used prospective case control study. Stroke patients with GHS were recruited and assigned to control group and FMS group. Control group received electrode stimulation at the supraspinatus and deltoid muscles of the hemiplegic side, while FMS group was stimulated at the same locations. Before and after treatment, the distances of the acromion-greater tuberosity (AGT), acromion-lesser tuberosity (ALT), acromiohumeral distance (AHD), supraspinatus thickness (SST), and deltoid muscle thickness (DMT) in patients' bilateral shoulder joint were measured by MSUS, respectively. Meanwhile, Fugl-Meyer Assessment (FMA) was used to evaluate the improvement of upper limb function.

**Results:**

30 patients were recruited. After FMS treatment, there was a significant decrease in the difference value between ipsilateral side and contralateral side of AGT [*t* = 8.595, *P* < 0.01], ALT [*t* = 11.435, *P* < 0.01], AHD [*t* = 8.375, *P* < 0.01], SST [*t* = 15.394, *P* < 0.01], and DMT [*t* = 24.935, *P* < 0.01], and FMA score increased [*t* = −13.315, *P* < 0.01]. Compared with control group, FMS group decreased more significantly in the difference value between ipsilateral side and contralateral side of AGT [*t* = 2.161, *P* < 0.05], ALT [*t* = 3.332, *P* < 0.01], AHD [*t* = 8.768, *P* < 0.01], SST [*t* = 6.244, *P* < 0.01], and the DMT [*t* = 3.238, *P* < 0.01], and FMA score increased more significantly in FMS group [*t* = 7.194, *P* < 0.01].

**Conclusion:**

The study preliminarily shows that the MSUS can objectively and dynamically evaluate the treatment effect of GHS in hemiplegic patients. Meanwhile, compared with control group, the FMS is more effective and has fewer side effects, and the long-term effect of FMS is worth further study. This trial is registered with ChiCTR1800015352.

## 1. Introduction

Glenohumeral subluxation (GHS) is common in patients with stroke and has an effect on the recovery of upper limb motor function [1–3]. The incidence of GHS has been reported from 17% to 84%; such a difference is mainly due to different measurement methods [4, 5]. Musculoskeletal ultrasonography (MSUS) plays an important role in the nervous system, orthopedics, and rehabilitation as an imaging model [5]. Its advantages include high accuracy, low cost, real-time imaging, contralateral immediate comparison, and radiation-free [6]. In recent years, it has replaced palpation and plain radiograph [7] and become the main means to assess shoulder abnormalities.

GHS can be assessed by measuring the distance between the acromion and the humerus. Kumar et al. have confirmed that MSUS measurement of acromion-greater tuberosity (AGT) distance is reliable and effective in assessing GHS in hemiplegic patients [2, 8]. Park et al. found that increased acromion-lesser tuberosity (ALT) distance was highly correlated with GHS [4]. Previous studies have demonstrated the validity and reliability of using MSUS to measure acromiohumeral distance (AHD) for assessing GHS [9–13]. Nozoe et al. have elucidated that the recovery of limbs function can be assessed by measuring changes in the thickness of dominated muscles before and after the treatments [14, 15]. Some researchers have confirmed the validity and accuracy of using MSUS to measure muscle thickness [16–18]. Therefore, we measured the change of AGT, ALT, AHD, and the supraspinatus thickness (SST) and deltoid muscles thickness (DMT) to evaluate the improvement of GHS and upper limb function before and after the treatments.

Among reasons for GHS, the denervation of shoulder muscles caused by brain injury is the main cause and, under the action of gravity, the humeral head downward out of the glenoid fossa [19]. Without timely and effective treatment, GHS will get worse over time and eventually become irreversible [20]. Thus, the treatment of GHS is suggested as early and effectively as possible [21]. A number of methods have been reported for the treatment of GHS, such as shoulder slings, shoulder strapping, positioning, and electrical stimulation [7, 22, 23]. Many studies have shown that functional electrical stimulation (FES) is effective for the treatment of GHS in acute hemiplegic patients [21, 22, 24, 25]. According to electromyographic studies, the supraspinatus and deltoid muscles are the two key muscles that maintain the head of the humerus in the glenoid fossa, so they are the target spots of the FES treatment [21, 22]. However, FES has some adverse reactions, for example, inducing pain, dermatitis, and even skin burns. In addition, the role of FES is very weak for the deep muscles and nerves, because its stimulating scope is shallow [26, 27]. And FES is invalid in chronic hemiplegic patients. Therefore, it is necessary to find an alternative method.

Functional magnetic stimulation (FMS) has been used to stimulate the muscles and peripheral nerves to promote function recovery. A lot of researches have confirmed the effectiveness of FMS in the aspects of gastric emptying, neurogenic bowel, respiratory muscle conditioning, dysphagia, urinary incontinence, and so on [28–32]. Okudera et al. have confirmed that FMS can improve upper limb motor function in healthy adults [27]. So we hypothesize that FMS may reduce GHS and promote the recovery of upper limb function in hemiplegic patients.

The purpose of our study was to, using MSUS, objectively quantify the effect of FMS treatment on GHS in hemiplegic patients with acute stroke. Compared with electrical stimulation, maybe FMS can achieve the same or even better therapeutic effects with lesser side effects.

## 2. Methods

### 2.1. Participants

Patients who were inpatient or outpatient at the Physical Medicine & Rehabilitation Department of the First Affiliated Hospital of Soochow University were screened from August 2016 to May 2017. The inclusion criteria were as follows: (1) stroke onset time of less than one month, (2) less than or equal to grade 3 of the upper limb muscle strength in hemiplegic side, (3) stable vital signs, (4) without aphasia or cognitive dysfunction, and (5) able to sit upright independently (or with one person's assistance). And exclusion criteria were as follows: (1) history of shoulder dysfunction, (2) combined with myogenic diseases or the peripheral nervous system disease, (3) combined with severe heart, liver, or kidney dysfunction, (4) pacemaker or metal implantation, and (5) combined with severe coagulation dysfunction.

The finger breadth palpation methods were used for GHS diagnosis; that is, AHD is 1/2 fingerbreadth or more. The degree of GHS is defined as follows: 0 degrees = no subluxation, 1 degree = 1/2 fingerbreadth gap, 2 degrees = 1 fingerbreadth gap, 3 degrees = 1 1/2 fingerbreadth gap, 4 degrees = 2 fingerbreadth gap, and 5 degree = 2 1/2 fingerbreadth gap [5, 33]. Patients with a dislocation of 2 degree or more were included in the study.

### 2.2. Study Design

The study is a prospective case control study. The recruited patients were assigned to control group and FMS group. Basic information including age, gender, duration of stroke, affected side, and type of stroke were collected from patients and their guardians. The informed consent was signed by patients themselves, and to those who were unable to sign, their guardians were authorized to sign. The study was approved by the Ethics Committee of the First Affiliated Hospital of Soochow University. Screening of eligible patients and collection of basic information were done by one person in the research team.

### 2.3. Treatments

Both groups received conventional rehabilitation including active and passive motion, weight bearing exercise, grasp, hold and release activities, and ADL activities, 45 minutes a day, consecutive 5 days a week, total for 4 weeks. The conventional rehabilitation of all patients was done by one therapist.

On the basis of conventional rehabilitation, control group received stimulation by electrode stimulation device (BA2008-III, Benao, China). Four electrodes were attached to the places of the supraspinatus and deltoid muscles of the hemiplegic side. And pulse of 200 and micro/s, duty cycle of 1 : 2, wave rise/wave drop of 2 s, and current of 50 mA were applied. The FMS group used magnetic stimulator (MagPro R30, Medtronic A/S, Denmark) connected with a 75 mm figure-of-eight water-cooled coil (MCF-B65) to stimulate the supraspinatus and deltoid muscles of the hemiplegic side. Frequency of 5 Hz with the stimulus intensity at 100% of the resting motion threshold (MT) was applied. Each site of each patient was stimulated for 20 minutes a day in both groups. The treatments were consecutively conducted 5 days a week for a total of 4 weeks. During the treatments, patients were seated upright independently (or with one person's assistance) in a chair with forearms placing on their laps. Two special therapists were, respectively, trained in one of the treatments, to complete the patients' treatment programs. In FMS group no patient withdrawn during the treatments, but in control group, there are 4 patients who failed to persist in electrical stimulation due to pain.

### 2.4. Clinical Evaluations

A portable diagnostic ultrasound system (M-Turbo, ICTx, SonoSite, America) connected with a 6–13 MHz linear array transducer was used to assess the changes of GHS, AGT, ALT, AHD, SST, and DMT, respectively, in the hemiplegic side and healthy side of shoulder before and after the treatments. At the same time, we used Fugl-Meyer Assessment (FMA) Scale to assess upper limb function of the hemiplegic side in acute poststroke patients.

In the process of measuring these parameters, each patient was placed in a standardized position [2]. Patients were seated upright in a chair with their forearms placed on their laps and the elbows unsupported. Each parameter was measured three times and took the average by the same specially trained sonographer before and after the treatments. The parameters were measured on the frozen image using the caliper on the screen. The depth of the ultrasonic transducer was set at 3.5 cm for each measurement.

**Figure 1:**
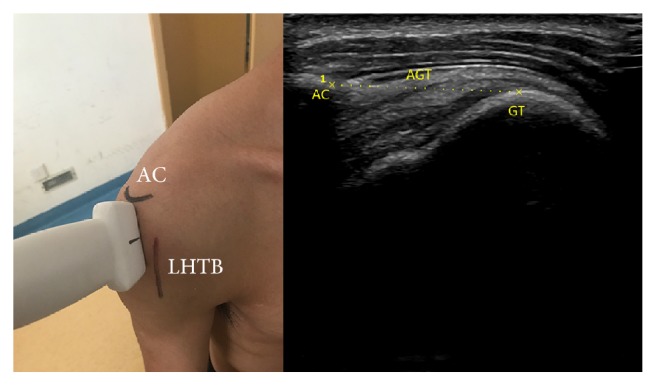
Anatomical structures: AC (acromion); LHTB (long head tendon of biceps); the position and orientation of transducer; and the ultrasound image: GT (greater tuberosity) and AGT (acromion-greater tuberosity).

*AGT*. Place transducer on the lateral edge of acromion and the lateral edge of long head tendon of biceps; scan the shoulder along longitudinal axis of humerus. When the lateral edge of acromion and the upper edge of greater tuberosity simultaneously appear in the screen freeze the image and measure AGT. We calculated the difference value of AGT between ipsilateral side and contralateral side and recorded (Figure 1).

**Figure 2:**
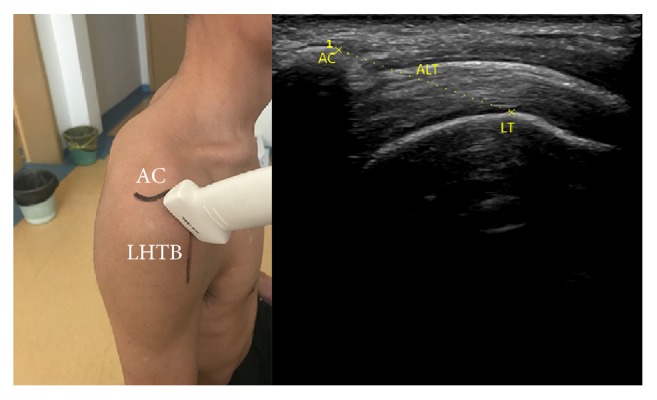
Anatomical structures; the position and orientation of transducer; and the ultrasound image: LT (lesser tuberosity) and ALT (acromion-lesser tuberosity).

*ALT*. Place transducer on the lateral edge of acromion and the medial edge of long head tendon of biceps; scan the shoulder along longitudinal axis of humerus. When the lateral edge of acromion and the upper edge of lesser tuberosity simultaneously appear in the screen freeze the image and measure ALT. We calculated the difference value of ALT between ipsilateral side and contralateral side and recorded (Figure 2).

**Figure 3:**
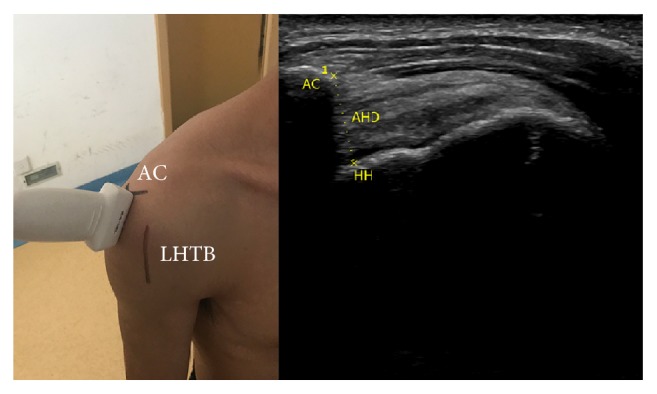
Anatomical structures; the position and orientation of transducer; and the ultrasound image: HH (humerus head) and AHD (acromiohumeral distance).

*AHD*. Place transducer on the anterior edge of acromion in the coronal plane. When acromion and humerus head simultaneously appear in the screen freeze the image and measure the shortest distance between acromion and humerus. We calculated the difference value of AHD between ipsilateral side and contralateral side and recorded (Figure 3).

**Figure 4:**
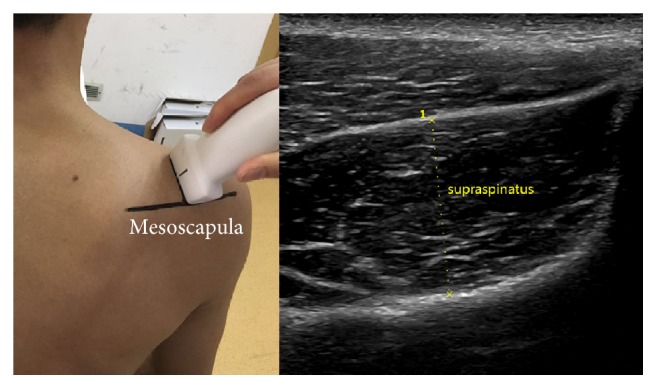
Anatomical structures: mesoscapula; the position and orientation of transducer; and the ultrasound image: SST (supraspinatus thickness).

*SST*. Place transducer vertically at the midpoint of the mesoscapula. Move transducer in parallel until identifying the thickest cross section of supraspinatus, freeze the image, and measure the distance of the thickest part of supraspinatus. We calculated the difference value of SST between ipsilateral side and contralateral side and recorded (Figure 4).

**Figure 5:**
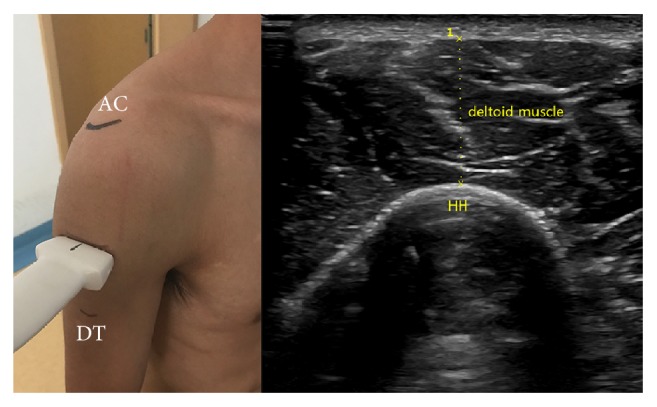
Anatomical structures: DT (deltoid tuberosity); the position and orientation of transducer; and the ultrasound image: DMT (deltoid muscle thickness).

*DMT*. We measure the thickness of the middle bundle representing DMT in our research. Place transducer vertically at the midpoint of the connection of acromion lateral edge and deltoid tuberosity. Move transducer in parallel until identifying the thickest cross section of deltoid muscle, freeze the image, and measure the distance of the thickest part of deltoid muscle. We calculated the difference value of DMT between ipsilateral side and contralateral side and recorded (Figure 5).

### 2.5. Statistical Analysis

Using SPSS19.0 software to do data statistics and analysis, the data were expressed as mean value ± standard deviation. *T* test was used to compare data and *P* < 0.05 was statistically significant.

## 3. Results

### 3.1. General Information

A total of 34 patients were evaluated and treated, and 4 patients who failed to persist in electrical stimulation due to pain were excluded. Finally, 30 patients (23 men, 7 women) with a mean age of 65 years (range from 38 to 84 years) were eligible for the study. The mean duration after onset was 15 days (range from 7 to 21 days). A summary of the demographic characteristics of the patients is shown in Table 1. There is no difference between the two groups with regard to age, gender, duration of stroke, affected side, type of stroke, and degree of GHS (*P* > 0.05).

### 3.2. The Results of MSUS Measurement

**Figure 6:**
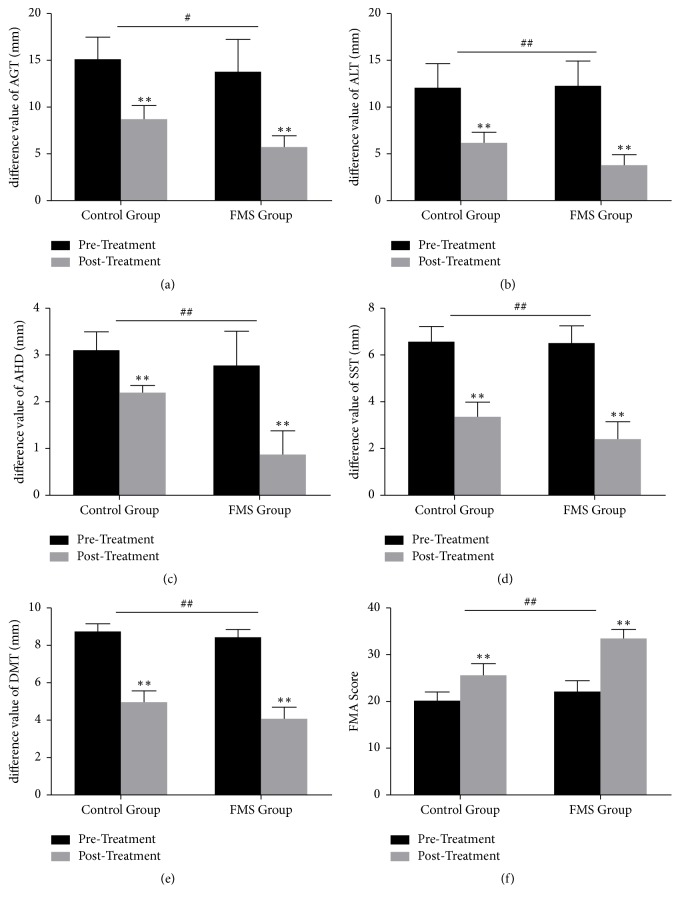
Comparison within control group and FMS group, respectively, and comparison between control group and FMS group. Comparison within groups: ^*∗*^*P* < 0.05 and ^*∗∗*^*P* < 0.01. Comparison between groups: ^#^*P* < 0.05 and ^##^*P* < 0.01.

Before treatments, there were no differences statistical significance (*P* > 0.05) in the value of AGT, ALT, AHD, SST, and DMT between control group and FMS group. After treatments, there was a significantly decrease in the difference value between ipsilateral side and contralateral side of AGT, ALT, AHD, SST, and DMT in both groups (Tables 2 and 3 and Figures 6(a)–6(d)). After 4-week treatment, compared with control group, FMS group decreased more significantly in the difference value between ipsilateral side and contralateral side of AGT, ALT, AHD, SST, and DMT (Table 4 and Figures 6(a)–6(d)).

### 3.3. The Results of FMA Assessments

We used the Simplified Fugl-Meyer Motor Function Assessment Scale to assess the hemiplegic upper limb function before and after the treatments, respectively. Before treatments, there was no differences statistical significance (*P* > 0.05) in FMA score between control group and FMS group. After the treatments, FMA score increased in both control group and FMS group (Tables 2 and 3 and Figure 6(f)). After 4-week treatment, compared with control group, FMA increased more significantly in FMS group (Table 4 and Figure 6(f)).

## 4. Discussion

In our study, the results of MSUS preliminary proved in FMS group, the gap between the ipsilateral side and contralateral side of AGT, ALT, and AHD significantly decreased, and SST and DMT obviously increased. At the same time, FMA substantially improved in the hemiplegic upper limb. These results show that the short time FMS treatment (4 weeks) can obviously improve GHS of the hemiplegic patients with acute stroke and promote the functional recovery of the patients' paralyzed upper limbs. The changes in several indicators by MSUS measurement, such as AGT, ALT, and AHD, as well as SST and DMT, are consistent with the function of paralyzed upper limb. These coincide with our previous assumptions.

Researches have shown that, in acute poststroke hemiplegic patients, paralysis muscles around the shoulder cannot resist the gravity of upper limb and gradually result in GHS [20, 23]. Soft tissue around the shoulder such as muscles, ligaments, and capsule is going to be overstretched, leading to the dysfunction of upper limb [2]. Therefore, our goal is to use simple and effective rehabilitation therapies to restore the activity of the paralyzed muscles, enhance its ability to resist gravity, reduce GHS, and ultimately achieve the recovery of upper limb function.

Electrical stimulation and magnetic stimulation techniques have been widely used in the field of rehabilitation. Our study results are consistent with previous studies that FES is effective on reducing GHS and promoting the recovery of upper limb function in acute hemiplegic patients [21, 22, 24, 25]. After electrical stimulation treatment in control group, the AGT and AHD of hemiplegic side were remarkably decreased and FMA increased obviously in our study. However, the mechanism has not yet been fully understood. Perhaps by the stimulation of the muscle fibers and peripheral nerves, the muscles contraction increases and the coordination between agonistic and antagonistic muscles improve and eventually achieve functional and beneficial movement [20, 25], although the FES is effective on GHS in acute (<6 months) hemiplegic patients but is not valid in chronic (>6 months) ones [26, 34]. Perhaps the soft tissue around the shoulder overstretched for too long time, and the muscles atrophy is very serious, so it induced the GHS quite difficult to recover. Several studies have also found that the GHS can hardly get further improved after 6 weeks of FES treatment [22, 35]. And follow-up studies have also shown that there is no a positive long-term effect after FES [21, 34]. Because of the limitations and complications in the skin and other aspects, its clinical application is limited.

While repeat transcranial magnetic stimulation (rTMS) is a kind of brain stimulation techniques which has become promising for the recovery of limbs function in hemiplegic patients [36], it stimulates the cerebral cortex to regulate the excitability of the central nervous system [37, 38]. Sohn et al. have confirmed that rTMS has a certain effect on the recovery of upper limb function in hemiplegic patients [36, 37]; however, there have some side effects to be reported, such as induced epilepsy, tinnitus, and headache [39]. In order to avoid these complications, more and more researchers have studied its use in stimulating peripheral nerves and muscles, and it is called functional magnetic stimulation (FMS).

The FMS has been applied in some aspects of rehabilitation and achieved some curative effects [28–32]. Since the FMS does not paste on the skin directly, it seldom causes skin problems. However, the applications of FMS in reducing GHS and promoting the recovery of upper limb function have not yet been reported. Inspired by previous researches, our team speculated that the FMS used in local hemiplegic shoulders maybe produce the same or even better effect as electrical stimulation. And this was confirmed in our study. After the treatments for 4 weeks, compared with control group the AGT and AHD in the hemiplegic side were significantly reduced and the FMA scores increased in FMS groups. These trends are more pronounced and coincide with our previous assumptions. Since the magnetic field of FMS does not attenuate through the skin, it can act on deeper muscles and peripheral nerves than electrical stimulation [40]. And it is painless and causes no damage to the skin. In our study, we found that pain and dermatitis appeared more or less in the stimulation area of patients in control group, which did not appear in FMS group.

Our previous studies have shown that MSUS measurement of AGT is reliable and valid in assessing GHS in patients with hemiplegia, which is in agreement with many other researchers [41]. A lot of researches are done by Bladel et al., and they found that it was valid and reliable to evaluate the therapeutic effect of GHS by measuring changes in AHD [9, 10]. McCreesh et al. have confirmed that MSUS is a reliable and sensitive tool to identify AHD change [10–13]. From our study it can be found the AGT, ALT, and AHD consistently reduced after the treatments, especially in FMS group. The results not only show that FMS is more effective than electrical stimulation, but also further illustrate the correlation between ALT and GHS. The greater tuberosity and the lesser tuberosity are adjacent, and the brachial biceps long head tendon goes through the gap between them. It is not difficult to measure ALT with MSUS. So, in addition to measuring AGT and AHD with MSUS, we also assess GHS by measuring ALT. It may be used as a supplement or even become a more sensitive indicator. Although it has been reported that ALT was highly correlated with GHS [4], its validity and reliability of assessing GHS by ALT alone need our further specialized researches to verify.

Muscle thickness affects muscle function and further affects limb function [15]. CT and MRI have been the standard methods for accurate measurement of muscle thickness [6, 18]. But it has shortcomings of radio action, cost too much, inconvenience, and so on. Nozoe et al. have pointed out that MSUS measurement of muscle thickness is an effective way to assess the limb function of hemiplegic patients [14]. Therefore, we considered whether it was possible to evaluate the effect of FMS on the recovery of upper limb function in GHS patients by measuring the SST and DMT by MSUS. Dupont et al. have verified that MSUS can be used to measure SST and DMT [18]. Our study shows that the thickness of the two muscles significantly increase in both groups, especially in FMS group. By stimulating the muscles and peripheral nerves, FMS and FES can, on the one hand, maintain muscles and peripheral nerves excitability and reduce disuse atrophy and, on the other hand, feedback regulate and remodel the corresponding functional areas of the cerebral cortex to restore muscle contraction. The FMS can act on deeper muscles and peripheral nerves than electrical stimulation, making FMS a better treatment.

In addition, previous studies have shown that the change of muscle thickness could be used as an indicator of muscle atrophy [18]. However, in this study the change of muscle thickness around the shoulder in hemiplegic patients is not only caused by muscle atrophy alone. Because the recruited patients were all at the early stage of hemiplegia, muscle atrophy was not obvious in a short time. We have reasons to believe that the more important reason is the paralyzed muscles around the shoulder which are elongated due to the separation of bone structure in GHS, making the muscles become thinner. Therefore, from this point of view, SST and DMT can also predict the extent of the GHS. Furth more, in both control group and FMS group, the FMA scores of upper limb function in hemiplegic side were significantly improved after the treatments. Compared with control group, it improved more significantly in FMS group. This is consistent with the results of MSUS, thus further verifying the relationship between each indicators of MSUS measurement and limb function. These indicators may be used as an objective basis for monitoring the degree of GHS.

Recently, some researchers have studied the synergistic effects of different therapies on limb function recovery after stroke and found that they were more pronounced than single treatment [7, 42]. So the next step, we will study the synergistic effects of FMS and some other therapies on GHS and upper limb function in hemiplegic patients.

Of course, our research also has many limitations. First, the sample size is not large enough. Second, the further follow-up is needed to assess the long-term effect. Third, whether FMS is effective in GHS in patients with chronic hemiplegia is not validated. These questions deserved further exploration and study.

## 5. Conclusion

The FMS is effective in the treatment of GHS in patients with early hemiplegia, and compared with the electrical stimulation, it is more effective. The mechanism, effectiveness in chronic hemiplegia, long-term efficacy, and so on of the new way, FMS, need further exploration. Meanwhile, the changes in several indicators by MSUS measurement, such as AGT, ALT, and AHD, as well as SST and DMT, are consistent with the function of paralyzed upper limb. These indicators may be used as an objective basis for monitoring the degree of GHS. Furthermore, the validity and reliability of ALT, SST, and DMT in assessing GHS and upper limb functional require further proof.

## Figures and Tables

**Table 1:** Patients' demographics.

Type of variable	Control group	FMS group	*P* value
Age (years)	67.20 ± 10.72	63.67 ± 15.09	0.146
Gender			0.418
Male	11.00	12.00	
Female	4.00	3.00	
Duration of stroke (days)	15.47 ± 2.72	13.87 ± 3.36	0.359
Affected side			0.224
Left	6.00	7.00	
Right	9.00	8.00	
Type of stroke			1.000
Hemorrhage	5.00	6.00	
Ischemia	10.00	9.00	
Degree of GHS			0.603
2 degrees	2.00	3.00	
3 degrees	11.00	11.00	
4 degrees	2.00	1.00	

**Table 2:** Comparison between posttreatment and pretreatment in control group.

	Pretreatment	Posttreatment	*t* value	*P* value
Difference value of AGT	15.05 ± 2.41	8.70 ± 1.43	8.766	0.000
Difference value of ALT	12.15 ± 2.55	6.22 ± 1.17	8.170	0.000
Difference value of AHD	3.11 ± 0.37	2.20 ± 0.15	8.762	0.000
Difference value of SST	6.61 ± 0.63	3.37 ± 0.64	14.010	0.000
Difference value of DMT	8.76 ± 0.39	5.00 ± 0.58	20.986	0.000
FMA score	20.00 ± 2.17	25.40 ± 2.69	6.045	0.000

**Table 3:** Comparison between posttreatment and pretreatment in FMS group.

	Pretreatment	Posttreatment	*t* value	*P* value
Difference value of AGT	13.75 ± 3.44	5.70 ± 1.15	8.595	0.000
Difference value of ALT	12.30 ± 2.64	3.93 ± 1.03	11.435	0.000
Difference value of AHD	2.78 ± 0.72	0.90 ± 0.49	8.375	0.000
Difference value of SST	6.54 ± 0.73	2.44 ± 0.73	15.394	0.000
Difference value of DMT	8.45 ± 0.36	4.13 ± 0.57	24.935	0.000
FMA score	22.00 ± 2.54	33.47 ± 2.17	13.315	0.000

**Table 4:** Comparison between FMS group and control group.

	FES	FMS	*t* value	*P* value
Difference value of AGT	6.35 ± 1.49	8.05 ± 2.66	2.161	0.039
Difference value of ALT	5.93 ± 2.21	8.37 ± 1.78	3.332	0.002
Difference value of AHD	0.91 ± 0.31	1.88 ± 0.29	8.768	0.000
Difference value of SST	3.24 ± 0.44	4.09 ± 0.29	6.244	0.000
Difference value of DMT	3.76 ± 0.60	4.32 ± 0.29	3.238	0.003
FMA score	5.40 ± 1.80	11.47 ± 2.72	7.194	0.000

## References

[1] Wang R. Y., Yang Y. R., Tsai M. W., Wang W. T., Chan R. C. (2002). Effects of functional electric stimulation on upper limb motor function and shoulder range of motion in hemiplegic patients. *American Journal of Physical Medicine & Rehabilitation*.

[2] Kumar P., Cruziah R., Bradley M., Gray S., Swinkels A. (2016). Intra-rater and inter-rater reliability of ultrasonographic measurements of acromiongreater tuberosity distance in patients with post-stroke hemiplegia. *Topics in Stroke Rehabilitation*.

[3] Paci M., Nannetti L., Taiti P., Baccini M., Rinaldi L. (2007). Shoulder subluxation after stroke: relationships with pain and motor recovery.. *Physiotherapy research international : the journal for researchers and clinicians in physical therapy*.

[4] Park G.-Y., Kim J.-M., Sohn S.-I., Shin I.-H., Lee M. Y. (2007). Ultrasonographic measurement of shoulder subluxation in patients with post-stroke hemiplegia. *Journal of Rehabilitation Medicine*.

[5] Kumar P., Mardon M., Bradley M., Gray S., Swinkels A. (2014). Assessment of glenohumeral subluxation in poststroke hemiplegia: Comparison between ultrasound and fingerbreadth palpation methods. *Physical Therapy in Sport*.

[6] English C. K., Thoirs K. A., Fisher L., McLennan H., Bernhardt J. (2012). Ultrasound is a reliable measure of muscle thickness in acute stroke patients, for some, but not all anatomical sites: a study of the intra-rater reliability of muscle thickness measures in acute stroke patients. *Ultrasound in Medicine & Biology*.

[7] Paci M., Nannetti L., Rinaldi L. A. (2005). Glenohumeral subluxation in hemiplegia: An overview. *Journal of Rehabilitation Research and Development *.

[8] Kumar P., Bradley M., Gray S., Swinkels A. (2011). Reliability and validity of ultrasonographic measurements of acromion-greater tuberosity distance in poststroke hemiplegia. *Archives of Physical Medicine and Rehabilitation*.

[9] Van Bladel A., Lambrecht G., Oostra K. M., Vanderstraeten G., Cambier D. (2017). Arandomized controlled trial on the immediate and long-term effects of arm slings on shoulder subluxation in stroke patients. *European Journal of Physical and Rehabilitation Medicine*.

[10] McCreesh K. M., Crotty J. M., Lewis J. S. (2015). Acromiohumeral distance measurement in rotator cuff tendinopathy: Is there a reliable, clinically applicable method? A systematic review. *British Journal of Sports Medicine*.

[11] Lin Y.-S., Boninger M. L., Day K. A., Koontz A. M. (2015). Ultrasonographic measurement of the acromiohumeral distance in spinal cord injury: Reliability and effects of shoulder positioning. *The Journal of Spinal Cord Medicine*.

[12] Desmeules F., Minville L., Riederer B., Côté C. H., Frémont P. (2004). Acromio-humeral distance variation measured by ultrasonography and its association with the outcome of rehabilitation for shoulder impingement syndrome. *Clinical Journal of Sport Medicine*.

[13] Kalra N., Seitz A. L., Boardman N. D., Michener L. A. (2010). Effect of posture on acromiohumeral distance with arm elevation in subjects with and without rotator cuff disease using ultrasonography. *Journal of Orthopaedic & Sports Physical Therapy*.

[14] Nozoe M., Kubo H., Furuichi A. (2017). Validity of Quadriceps Muscle Thickness Measurement in Patients with Subacute Stroke during Hospitalization for Assessment of Muscle Wasting and Physical Function. *Journal of Stroke and Cerebrovascular Diseases*.

[15] Liu P., Wang Y., Hu H., Mao Y., Huang D., Li L. (2014). Change of muscle architecture following body weight support treadmill training for persons after subacute stroke: Evidence from Ultrasonography. *BioMed Research International*.

[16] Schneebeli A., Egloff M., Giampietro A., Clijsen R., Barbero M. (2014). Rehabilitative ultrasound imaging of the supraspinatus muscle: Intra- and interrater reliability of thickness and cross-sectional area. *Journal of Bodywork and Movement Therapies*.

[17] Yi T. I., Han I. S., Kim J. S., Jin J. R., Han J. S. (2012). Reliability of the supraspinatus muscle thickness measurement by ultrasonography. *Annals of Rehabilitation Medicine*.

[18] Dupont A.-C., Sauerbrei E. E., Fenton P. V., Shragge P. C., Loeb G. E., Richmond F. J. R. (2001). Real-time sonography to estimate muscle thickness: Comparison with MRI and CT. *Journal of Clinical Ultrasound*.

[19] Huang S.-W., Liu S.-Y., Tang H.-W., Wei T.-S., Wang W.-T., Yang C.-P. (2012). Relati onship between severity of shoulder subluxati on and soft-tisue injury in hemiplegic stroke patients. *Journal of Rehabilitation Medicine*.

[20] Manigandan J. B., Ganesh G. S., Pattnaik M., Mohanty P. (2014). Effect of electrical stimulation to long head of biceps in reducing gleno humeral subluxation after stroke. *NeuroRehabilitation*.

[21] Ada L., Foongchomcheay A. (2002). Efficacy of electrical stimulation in preventing or reducing subluxation of the shoulder after stroke: a meta-analysis. *Australian Journal of Physiotherapy*.

[22] Stolzenberg D., Siu G., Cruz E. (2012). Current and future interventions for glenohumeral subluxation in hemiplegia secondary to stroke. *Topics in Stroke Rehabilitation*.

[23] Chatterjee S., Hayner K. A., Arumugam N. (2016). The california tri-pull taping method in the treatment of shoulder subluxation after stroke: A randomized clinical trial. *North American Journal of Medical Sciences*.

[24] Gu P., Ran J. J. (2016). Electrical Stimulation for Hemiplegic Shoulder Function: A Systematic Review and Meta-Analysis of 15 Randomized Controlled Trials. *Archives of Physical Medicine and Rehabilitation*.

[25] Koyuncu E., Nakipoğlu-Yüzer G. F., Doğan A., Özgirgin N. (2010). The effectiveness of functional electrical stimulation for the treatment of shoulder subluxation and shoulder pain in hemiplegic patients: a randomized controlled trial. *Disability and Rehabilitation*.

[26] Lee J., Baker L. L., Johnson R. E., Tilson J. K. (2017). Effectiveness of neuromuscular electrical stimulation for management of shoulder subluxation post-stroke: a systematic review with meta-analysis. *Clinical Rehabilitation*.

[27] Okudera Y., Matsunaga T., Sato M. (2015). The impact of high-frequency magnetic stimulation of peripheral nerves: Muscle hardness, venous blood flow, and motor function of upper extremity in healthy subjects. *Biomedical Research (Japan)*.

[28] Lin V. W.-H., Kim K. H., Hsiao I., Brown W. (2002). Functional magnetic stimulation facilitates gastric emptying. *Archives of Physical Medicine and Rehabilitation*.

[29] Tsai P.-Y., Wang C.-P., Chiu F.-Y., Tsai Y.-A., Chang Y.-C., Chuang T.-Y. (2009). Efficacy of functional magnetic stimulation in neurogenic bowel dysfunction after spinal cord injury. *Journal of Rehabilitation Medicine*.

[30] Lin V. W., Hsiao I. N., Zhu E., Perkash I. (2001). Functional magnetic stimulation for conditioning of expiratory muscles in patients with spinal cord injury. *Archives of Physical Medicine and Rehabilitation*.

[31] Momosaki R., Abo M., Watanabe S., Kakuda W., Yamada N., Mochio K. (2014). Functional magnetic stimulation using a parabolic coil for dysphagia after stroke. *Neuromodulation: Technology at the Neural Interface*.

[32] Chandi D. D., Groenendijk P. M., Venema P. L. (2004). Functional extracorporeal magnetic stimulation as a treatment for female urinary incontinence: 'The chair'. *BJU International*.

[33] Hall J., Dudgeon B., Guthrie M. (1995). Validity of clinical measures of shoulder subluxation in adults with poststroke hemiplegia.. *The American journal of occupational therapy. : official publication of the American Occupational Therapy Association*.

[34] Vafadar A. K., Côté J. N., Archambault P. S. (2015). Effectiveness of functional electrical stimulation in improving clinical outcomes in the upper arm following stroke: a systematic review and meta-analysis. *BioMed Research International*.

[35] Wang R.-Y., Chan R.-C., Tsai M.-W. (2000). Functional electrical stimulation on chronic and acute hemiplegic shoulder subluxation. *American Journal of Physical Medicine & Rehabilitation*.

[36] Sohn Y. H., Hallett M. (2004). Motor evoked potentials. *Physical Medicine and Rehabilitation Clinics of North America*.

[37] Izumi S.-I., Kondo T., Shindo K. (2008). Transcranial magnetic stimulation synchronized with maximal movement effort of the hemiplegic hand after stroke: A double-blinded controlled pilot study. *Journal of Rehabilitation Medicine*.

[38] Inukai Y., Saito K., Sasaki R. (2016). Comparison of three non-invasive transcranial electrical stimulation methods for increasing cortical excitability. *Frontiers in Human Neuroscience*.

[39] Khedr E. M., Ahmed M. A., Fathy N., Rothwell J. C. (2005). Therapeutic trial of repetitive transcranial magnetic stimulation after acute ischemic stroke. *Neurology*.

[40] Paquette C., Thiel A. (2012). Rehabilitation interventions for chronic motor deficits with repetitive transcranial magnetic stimulation. *Journal of Neurosurgical Sciences*.

[41] Feng J., Yang W., Su M., Liu C., Zhang D. (2015). Reliability and validity of ultrasound measurement of acromion-greater tuberosity distance for assessing glenohumeral subluxation in hemiplegic patients. *Chinese Journal of Rehabilitation Medicine*.

[42] Etoh S., Noma T., Takiyoshi Y. (2016). Effects of repetitive facilitative exercise with neuromuscular electrical stimulation, vibratory stimulation and repetitive transcranial magnetic stimulation of the hemiplegic hand in chronic stroke patients. *International Journal of Neuroscience*.

